# Circulating Long Noncoding RNAs Act as Diagnostic Biomarkers in Non-Small Cell Lung Cancer

**DOI:** 10.3389/fonc.2020.537120

**Published:** 2020-12-07

**Authors:** Shuai Yuan, Ying Xiang, Xiaoping Guo, Yao Zhang, Chengying Li, Weijia Xie, Na Wu, Long Wu, Tongjian Cai, Xiangyu Ma, Zubin Yu, Li Bai, Yafei Li

**Affiliations:** ^1^ Department of Epidemiology, College of Preventive Medicine, Army Medical University (Third Military Medical University), Chongqing, China; ^2^ Center for Evidence-Based and Translational Medicine, Zhongnan Hospital of Wuhan University, Wuhan, China; ^3^ Department of Epidemiology, School of Public Health, Guizhou Medical University, Guiyang, China; ^4^ Department of Thoracic Surgery, Xinqiao Hospital, Army Medical University (Third Military Medical University), Chongqing, China; ^5^ Department of Respiratory Disease, Xinqiao Hospital, Army Medical University (Third Military Medical University), Chongqing, China

**Keywords:** circulating, long non-coding RNA, diagnosis, non-small cell lung cancer, biomarker

## Abstract

Identification of novel effective early diagnostic biomarkers may provide alternative strategies to reduce the mortality for non-small cell lung cancer (NSCLC) patients. Circulating long non-coding RNAs (lncRNAs) have emerged as a new class of promising cancer biomarkers. Our study aimed to identify circulating lncRNAs for diagnosing NSCLC. A total 528 plasma samples were continuously collected and allocated to four progressive phases: discovery, training, verification, and expansion phases. The expression of candidate lung cancer related lncRNAs were detected using quantitative reverse-transcriptase polymerase chain reaction (qRT-PCR). We identified a 4-lncRNA panel (RMRP, NEAT1, TUG1, and MALAT1) that provided a high diagnostic value in NSCLC (AUC = 0.86 and 0.89 for training and verification phase, respectively). Subgroup analyses showed that the 4-lncRNA panel had a sensitivity of 78.95% [95% confidence interval (CI) = 62.22%–89.86%] in stage I-II patients and 75.00% (95% CI = 52.95%–89.40%) in patients with small tumor size (≤3cm). Notably, the sensitivity of 4-lncRNA panel was significantly higher than that of routine protein panels in adenocarcinoma (CEA, CA125, and CYFRA21-1, 86.30% vs. 73.96%). Adding 4-lncRNA to protein markers significantly improved the diagnostic capacity in both adenocarcinoma (AUC=0.85, 95% CI = 0.78–0.91) and squamous cell carcinoma (AUC=0.93, 95% CI = 0.86–0.97). In conclusion, we identified a plasma 4-lncRNA panel that has considerable clinical value in diagnosing NSCLC. The 4-lncRNA panel could improve the diagnostic values of routine tumor protein markers in diagnosing NSCLC. Circulating lncRNAs could be used as promising candidates for NSCLC diagnosis.

## Introduction

Lung cancer is one of the most malignant tumors with high incidence and mortality in China and around the world (1, 2). Non-small cell lung cancer (NSCLC) is the major histological type, accounting for about 85% of lung cancer (3). Although NSCLC patients at early stages have a relatively high survival rate with optimized treatment, more than 75% of patients are diagnosed at advanced stages (4). The 5-year survival rate of NSCLC patients is still less than 20% due to the scarcity of effective early detection method (4).

Early detection and treatment is one of the most effective ways to improve curative effect and reduce mortality for NSCLC patients. Early detection method should be non-invasive and easily accessible (5). Low-dose computed tomography (LDCT) screening is recommended for the early detection of lung cancer, which can reduce lung cancer mortality by 20% (6). However, the false-positive rate of LDCT is relatively high (7). Moreover, high cost and repeated scanning have limited the application of LDCT (7). Circulating tumor protein markers, such as carcinoembryonic antigen (CEA), squamous cell carcinoma antigen (SCC), cytokeratin 19 fragment antigen (CYFRA21-1), can also act as noninvasive biomarkers to improve early diagnosis of NSCLC (8). However, the diagnostic performance of the protein markers for the early detection of NSCLC were limited because of unsatisfied sensitivity and specificity (8). Thus, identification of novel effective early diagnostic markers may provide alternative strategies to reduce the mortality for NSCLC patients.

Long non-coding RNAs (lncRNAs) have been proved to play important roles in occurrence and progression of many diseases, including NSCLC (9, 10). Studies have found a variety of abnormal expressed lncRNAs, which have important biological function in the process of NSCLC. Moreover, lncRNAs can be stably detected in the peripheral circulation. These features make circulating lncRNAs ideal noninvasive biomarkers for lung cancer diagnosis (11). In fact, differential expression of several circulating lncRNAs, including MALAT1 (12), GAS5 (13), SNHG1 (14), TUG1 (15), and HOTAIR (16) in patients with NSCLC were reported recently. Although these circulating lncRNAs have the ability to distinguish lung cancer patients from non-lung cancer patients, several challenges must be overcome to further develop circulating lncRNA-based biomarkers for clinical applications. The sample size in most of the current studies are relatively small and the results have not been verified in multiple stage clinical studies. In addition, few studies investigated circulating lncRNA for early detection of NSCLC patients and simultaneously compared the diagnostic performance of circulating lncRNAs with the routine tumor protein biomarkers.

In the present study, we investigated the diagnostic value of circulating lncRNAs in multiple progressive phases (discovery, training, and verification phases) to identify a panel of lncRNAs for the diagnosis of NSCLC. We also compared of the diagnostic performance of the lncRNAs panel with protein tumor markers in lung adenocarcinoma and squamous cell carcinoma, respectively.

## Materials and Methods

### Patient Samples and Study Design

Participants were continuously recruited from November 2015 to December 2017 at the Xinqiao Hospital Affiliated to Army Medical University in Chongqing, China. A total of 528 participants were enrolled and comprised of patients with newly diagnosed and histopathologically confirmed primary NSCLC, chronic obstructive pulmonary disease (COPD), pulmonary tuberculosis, pulmonary inflammation, other benign lung disease, and healthy controls. Blood samples from patients were collected prior to any treatment under fasting conditions.

Demographic and clinicopathological characteristics were obtained from all participants *via* a combination of a structured questionnaire and medical records. The serum concentrations of tumor markers (CEA, CA125, CYFRA21-1, SCC, and NSE) prior to any treatment were also collected. Research protocol was reviewed and approved by the ethics committee of the Army Medical University (Chongqing, China), and all participants provided informed consent.

**Figure 1:**
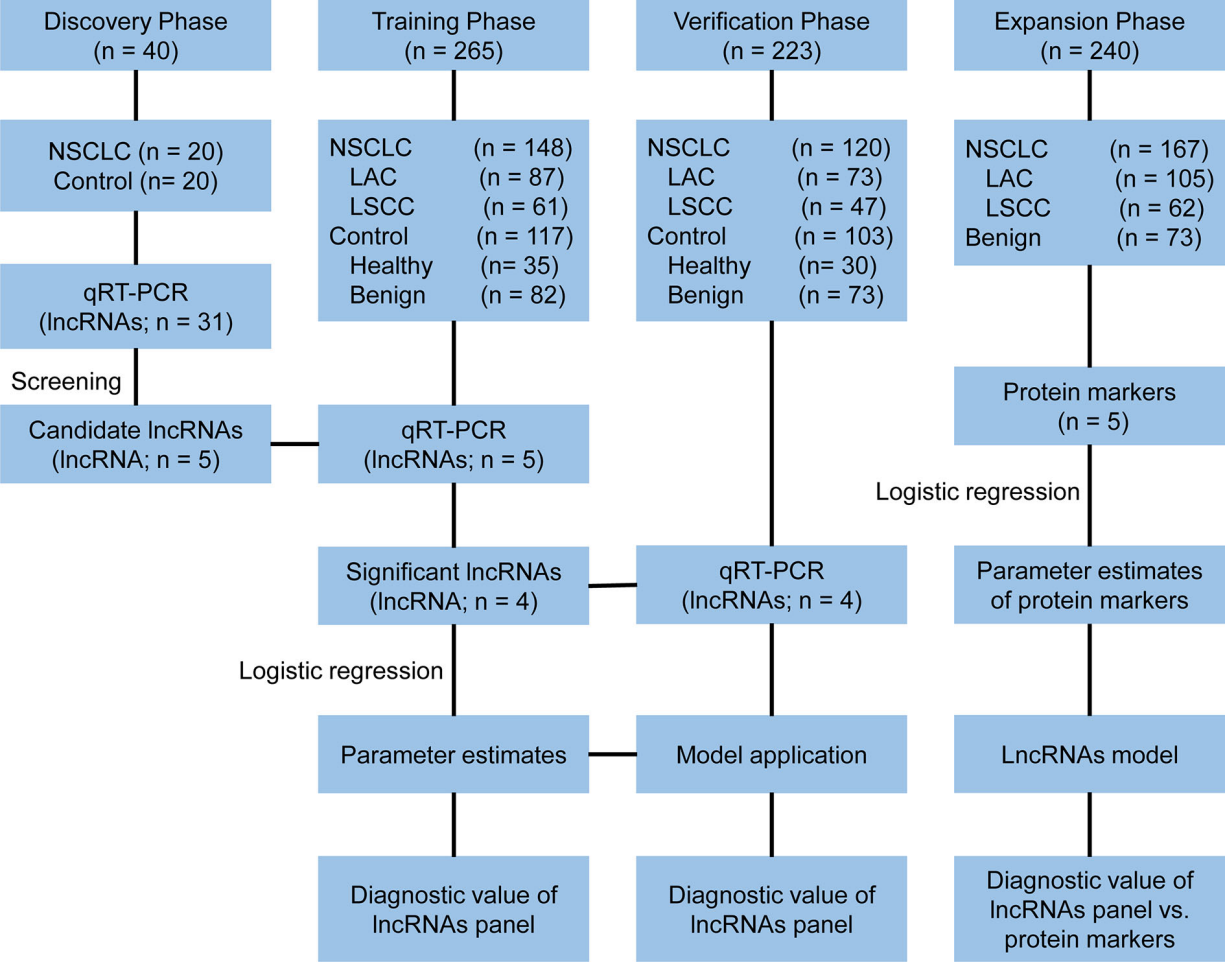
Study design. NSCLC, non-small cell lung cancer; LAC, lung adenocarcinoma; LSCC, lung squamous cell carcinoma; qRT-PCR, quantitative reverse transcriptase polymerase chain reaction.

A multi-phase study was designed to identify a panel of plasma lncRNA biomarkers. The study comprised four phases: the discovery phase, the training phase, the verification phase and the expansion phase (**Figure 1**). In the discovery phase, a total of 31 candidate lung cancer related lncRNAs were selected as potential diagnostic biomarkers according to previous studies and LncRNADisease database (**Supplementary Table S1**). The expression of 31 lncRNAs were detected with quantitative reverse transcriptase polymerase chain reaction (qRT-PCR) in 40 plasma samples (20 NSCLC patients and 20 controls). In the training phase, the stably expressed lncRNAs in the discovery phase were firstly detected with qRT-PCR in an independent cohort of plasma samples from 265 participants. LncRNAs that were differentially expressed between NSCLC and control groups (healthy and benign controls) were used to construct the diagnostic model. In the verification phase, the diagnostic performance of the lncRNAs panel from the training phase were verified in an independent cohort of 223 plasma samples. In the expansion phase, diagnostic models for lung adenocarcinoma and squamous cell lung carcinoma were constructed using the five tumor protein markers (CEA, CA125, CYFRA21-1, SCC, and NSE) in 240 participants. Comparisons of the diagnostic performance were conducted between the lncRNA-based model and tumor protein marker-based model in lung adenocarcinoma and squamous cell lung carcinoma, respectively.

### Plasma Processing, RNA Isolation, and qRT-PCR Analysis

All blood samples from patients were collected prior to any treatment under fasting conditions. Blood samples were processed to separate plasma within 2 h from collection by centrifugation (2,000 g for 10 min at 4°C, 12,000 g for 10 min at 4°C). Plasma samples were transferred to RNase/DNase-free tubes and stored at −80°C awaiting total RNA extraction. Total RNA from plasma was extracted using the TRIzol LS reagent (Invitrogen, Carlsbad, CA, USA) following the manufacturer’s instructions.

For qRT-PCR analysis, 7 μl total RNA from plasma was firstly reverse transcribed into complementary DNA using PrimeScript™ RT reagent kit with gDNA eraser (TaKaRa, Dalian, China) as follows: 37°C for 15 min, followed by 85°C for 5 s. Then, real-time PCR was performed using the SYBR Premix Ex Taq (TaKaRa) with the thermocycling conditions as follows: 95°C for 30 s, followed by 40 cycles of 95°C for 5 s, 60°C for 30 s, and 72°C for 30 s, followed by a final cycle of 72°C for 2 min. Results were normalized to the expression levels of *β*-actin as described previously (17, 18). Primers sequences are provided in **Supplementary Table S1**. The qRT-PCR results were calculate using 2^−△△Cq^ method.

### Detection of Tumor Markers

Serum CEA, CA125 and CYFRA21-1 levels were detected by chemiluminescence method using Roche reagent sets (Roche Diagnostics, Shanghai, China) following the manufacturer’s instructions. Serum SCC and NSE were determined by chemiluminescence method using Abbott reagent sets (Abbott, Chicago, USA) following the manufacturer’s instructions.

### Statistical Analysis

The relative expression levels of lncRNAs were expressed as median (quartile spacing) [M (P25, P75)] and the expression difference were evaluated using the Mann-Whitney U test using SPSS 19.0 software (SPSS, Inc., Chicago, IL, USA). The lncRNAs expression differences under different freeze-thaw cycles and different room temperature incubation times were evaluated by one-way repeated measures analysis of variance using SPSS 19.0 software. ROC curves were generated using MedCalc 19.0.7 (Med-Calc, Mariakerke, Belgium) and the area under the curves (AUC) were compared by the DeLong test. The Clinical Calculator online tool (http://vassarstats.net/clin1.html?tdsourcetag=s_pctim_aiomsg) was used to calculate the sensitivity, specificity, positive predictive value, negative predictive value, positive likelihood ratio, and negative likelihood ratio. A two-sided P-value less than 0.05 was taken as statistically significant.

## Results

### Patient Characteristics

A total of 528 participants were enrolled into our study. These participants were randomly allocated to a discovery phase (n = 40), a training phase (n = 265) and a verification phase (n = 223; **Figure 1**). The characteristics of the study participants were summarized in **Table 1**. A total of 20 NSCLC patients (including 12 adenocarcinoma and 8 squamous cell carcinoma) and 20 controls (including 5 healthy controls and 15 benign lung disease patients) were included in the discovery phase. Subsequently, 148 NSCLC patients (including 87 adenocarcinomas and 61 squamous cell carcinoma) and 117 controls (including 35 healthy controls and 82 benign lung disease patients) were included in the training phase. The verification phase comprised 120 NSCLC patients (including 73 adenocarcinomas and 47 squamous cell carcinoma) and 103 controls (including 30 healthy controls and 73 benign lung disease patients). Among the above mentioned 528 participants, a total of 240 participants who received the full panel of tumor marker test including CEA, CA125, CYFRA21-1, SCC, and NSE before therapy were allocated to an expansion phase to compare the diagnostic performance of the lncRNAs with tumor protein markers (**Figure 1**).

**Table 1:** Characteristics of study participants in the discovery, training, and verification datasets.

Characteristics	Discovery Phase		*χ2*	*P*	Training Phase		*χ2*	*P*	Verification Phase		*χ2*	*P*
	NSCLC (n=20)	Control (n=20)			NSCLC (n=148)	control (n=117)			NSCLC (n=120)	control (n=103)		
**Age at diagnosis (%)**							0.004	0.95				
≤60	13	9	1.616	0.204	79 (53.4)	62(53.0)			73(60.8)	59(57.3)	0.289	0.587
>60	7	11			69 (46.6)	55(47.0)			47(39.2)	44(42.7)		
**Gender (%)**							0.670	0.408				
Male	17	11	4.286	0.038	108(73.0)	80(68.4)			81(67.5)	68(66.0)	0.055	0.824
Female	3	9			40(23.0)	37(31.6)			39(32.5)	35(34.0)		
**Smoking history (%)**												
Current smoker	8	6	5.825	0.054	65(43.9)	64(54.7)	3.324	0.186	52(43.3)	52(50.5)	1.169	0.561
Ever smoker	8	3			32(21.6)	18(15.4)			21(17.5)	15(14.6)		
Never-smoker	4	11			51(34.5)	35(29.9)			47(39.2)	36(35.0)		
**Histological type**												
LAD	12				87(58.8)	—			73(60.8)	—		
LSCC	8				61(41.2)	—			47(39.2)	—		
**Tumor size**												
≤ 3cm	5				42(27.7)	—			32(26.7)	—		
> 3cm	15				76(52.0)	—			65(54.2)	—		
Not available	—				30(20.3)	—			23(19.2)	—		
**Lymph node metastasis**												
Positive	11				82(55.4)	—			62(51.7)	—		
Negative	9				50(33.8)	—			49(40.8)	—		
Not available	—				16(10.8)	—			9( 7.5)	—		
**Distant metastases**												
Positive	8				63(42.6)	—			41(34.2)	—		
Negative	12				78(52.7)	—			73(60.8)	—		
Not available	—				7( 4.7)	—			6( 5.0)	—		
**TNM stage**												
I+II	10				65(43.9)	—			56(46.7)	—		
III+IV	10				76(51.4)	—			59(49.2)	—		
Not available	—				7( 4.7)	—			5 ( 4.1)	—		
**Control groups**												
Healthy control		5			—	35(29.7)			—	30(29.1)		
COPD		4			—	38(32.2)			—	26(25.2)		
Pulmonary tuberculosis		8			—	16(13.6)			—	20(19.4)		
Pulmonary inflammation		3			—	23(19.5)			—	24(23.3)		
Other benign lung disease		—			—	6( 5.1)			—	3(2.9)		

NSCLC, non-small cell lung cancer; LAC, lung adenocarcinoma; LSCC, lung squamous cell carcinoma; COPD, chronic obstructive pulmonary disease.

### LncRNAs Screening

**Figure 2:**
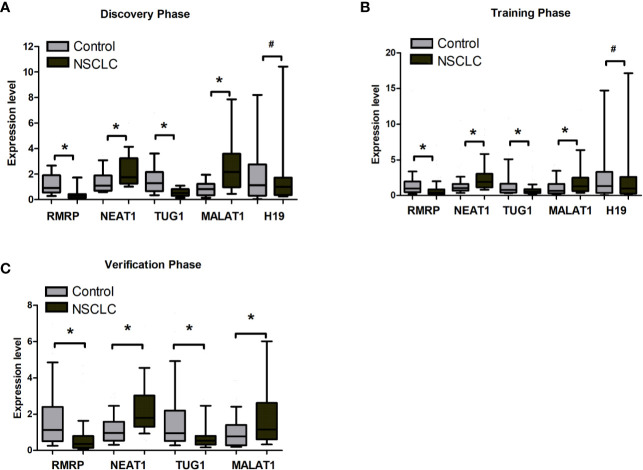
The expression level of RMRP, NEAT1, TUG1, MALAT1, and H19 between tumor and control plasma samples. **(A)** Discovery phase. **(B)** Training phase. **(C)** Verification phase. *Mann-Whitney U test, *P* < 0.05; ^#^Mann-Whitney U test, *P > 0.05.

By reviewing previous studies and LncRNADisease database, we selected 31 lung cancer related lncRNAs as potential diagnostic candidates (**Supplementary Table S1**). qRT-PCR were conducted to quantify the expression levels of 31 lncRNAs in 40 plasma samples (20 NSCLC patients and 20 controls). Five lncRNAs (RMRP, NEAT1, TUG1, MALAT1, and H19) stably expressed in plasma. Differential expression analysis showed that RMRP and TUG1 had significantly lower expression levels in the NSCLC group than in the control group (*P* = 0.001, **Figure 2A**, **Supplementary Table S2**). In contrast, NEAT1 and MALAT1 had significantly higher expression levels in the NSCLC group than in the control group (*P* = 0.022 and 0.002, respectively, **Figure 2A**, **Supplementary Table S2**). The expression level of H19 showed no significant difference between NSCLC and control groups (*P* = 0.534, **Figure 2A**, **Supplementary Table S2**).

The stability of RMRP, NEAT1, TUG1, MALAT1, and H19 in plasma was evaluated under harsh conditions. The expression levels of RMRP, NEAT1, TUG1, MALAT1, and H19 in plasma were detected after treating with repetitive multiple freeze-thaw cycles (1, 3, 5, and 8) or incubating for various durations (0, 4, 12, and 24 h) at room temperature. One-way repeated measures analysis of variance showed no significant difference in the expression levels of lncRNAs among freeze-thaw or incubating groups (*P* > 0.05) (**Supplementary Figure S1A, B**). In summary, the 5 stably expressed lncRNAs were identified as candidates for further testing in the training phase.

### Determination of the Diagnostic Value of the 4-lncRNA Panel in the Training Phase

The 5 lncRNAs were detected using an independent cohort of 265 plasma samples (including 148 NSCLC and 117 control) with qRT-PCR in the training phase. Four (RMRP, NEAT1, TUG1, and MALAT1) of the 5 lncRNAs had significantly different expression levels between the NSCLC and control groups, which were consistent with the results in the discovery phase (**Figure 2B**, **Supplementary Table S3**). Thus, RMRP, NEAT1, TUG1, and MALAT1 were selected as the final candidates for constructing a diagnostic model.

**Figure 3:**
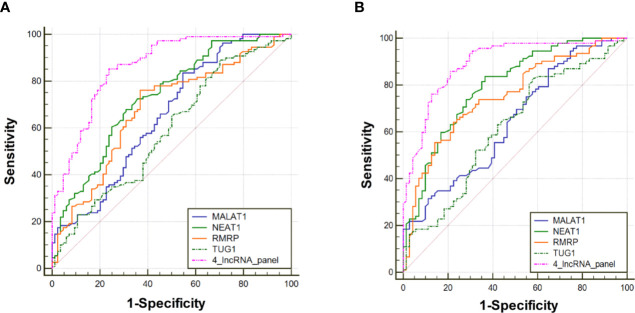
Receiver operating characteristic curve analysis for the diagnosis values of the 4-lncRNA panel and each lncRNA marker. **(A)** Training phase. **(B)** Verification phase.

The diagnostic value of RMRP, NEAT1, TUG1, and MALAT1 were firstly measured by ROC curves, which demonstrated a good discriminative ability between NSCLC and control groups (AUC = 0.70, 0.73, 0.65, and 0.66, respectively) **(** **Table 2**, **Figure 3A**). Then, a predictive lncRNAs panel was established by a stepwise logistic regression model using the training phase samples. All of the four lncRNAs turned out to be significant predictors. The predicted probability of the 4-lncRNA panel was calculated using following formula: Logit (P) =−1.083×RMRP+0.955×NEAT1−0.594×TUG1+0.530×MALAT1. The diagnostic performance of the established 4-lncRNA panel was evaluated by using ROC analysis, and the AUC for the 4-lncRNA panel was 0.86 [95% CI = 0.81 to 0.91; at a cut-off of 0.679, sensitivity = 85.32%, specificity = 76.19%, **Table 2**, **Figure 3A**]. The AUC value of the 4-lncRNA panel was significantly higher than that of any lncRNA alone (**Table 2**, *P* < 0.05).

**Table 2:** The diagnosis value of the lncRNAs and 4-lncRNA panel in the training dataset.

Markers	AUC(95% CI)	Cut-off	Youden index	Sensitivity (%)(95% CI)	Specificity (%)(95% CI)	+LR(95% CI)	-LR(95% CI)	PPV (%)(95% CI)	NPV (%)(95% CI)
RMRP	0.70 (0.64–0.75)^#^	≤0.660	0.41	72.03 (63.80–79.05)	68.97 (59.61–77.05)	2.32 (1.74–3.10)	0.41 (0.31–0.53)	74.10 (65.85–80.98)	66.67 (57.40–74.85)
NEAT1	0.73 (0.67–0.78)^#^	>1.338	0.37	67.91 (59.22–75.56)	68.97 (59.61–77.05)	2.19 (1.63–2.94)	0.47 (0.36–0.60)	71.65 (62.85–79.12)	65.04 (55.86–73.26)
TUG	0.65 (0.58–0.71)^#^	≤0.959	0.25	79.51 (71.05–86.06)	45.79 (36.22–55.67)	1.47 (1.21–1.78)	0.45 (0.31–0.65)	62.58 (54.42–70.11)	66.22 (54.19–76.55)
MALAT1	0.66 (0.60–0.72)^#^	>0.507	0.29	82.86 (75.36–88.50)	45.74 (35.54–56.30)	1.52 (1.25–1. 87)	0.37 (0.25–0.55)	71.37 (65.04–76.98)	69.47 (61.79–76.22)
4-lncRNA panel	0.86 (0.81–0.91)	>0.679	0.62	85.32 (76.96–91.12)	76.19 (65.42–84.52)	3.58 (2.43–5.29)	0.19 (0.12–0.31)	82.30 (73.75–88.60)	80.00 (69.26–87.80)

^#^The area under the curves of each lncRNA marker was compared with 4-lncRNA panel using the DeLong test with P<0.05. AUC, area under the curve; +LR, positive likelihood ratio; -LR, negative likelihood ratio; PPV, positive predictive value; NPV, negative predictive value; CI, confidence interval.

### Evaluation of the Diagnostic Performance of the 4-lncRNA Panel in the Verification Phase

To further assess the diagnostic value of the 4-lncRNA panel, we detected the 4 lncRNAs expression levels in another independent cohort of 223 plasma samples (including 120 NSCLC and 103 control) in the verification phase. The expression levels of the 4 lncRNAs were significantly different between patients with lung cancer and controls, which were consistent with the results in the training phase (**Figure 2C**, **Supplementary Table S4**). Similarly, the predicted AUC of the 4-lncRNA panel was 0.89 (95% CI = 0.84 - 0.94; at a cut-off of 0.679, sensitivity = 86.96%, specificity = 74.65%, **Table 3**, **Figure 3B**).

**Table 3:** The diagnosis value of the lncRNAs and 4-lncRNA panel in the verification dataset.

Markers	AUC(95% CI)	Cut-off	Youden index	Sensitivity (%)(95% CI)	Specificity (%)(95% CI)	+LR(95% CI)	-LR(95% CI)	PPV (%)(95% CI)	NPV (%)(95% CI)
RMRP	0.76 (0.70–0.82)^#^	≤0.660	0.34	69.50 (60.24–77.46)	64.70 (54.56–73.74)	1.97 (1.48–2.63)	0.47 (0.36–0.63)	69.50 (60.24–77.46)	64.70 (54.56–73.74)
NEAT1	0.78 (0.72–0.84)^#^	>1.338	0.43	72.90 (63.30–80.82)	69.60 (59.60–78.12)	2.40 (1.75–3.29)	0.39 (0.28–0.54)	71.56 (61.99–79.59)	71.00 (60.94–79.42)
TUG	0.67 (0.60–0.75)^#^	≤0.959	0.29	80.39 (71.11–87.34)	48.96 (38.69–59.31)	1.58 (1.27–1.96)	0.40 (0.26–0.61)	62.60 (53.67–70.77)	70.14 (57.57–80.40)
MALAT1	0.66 (0.58–0.74)^#^	>0.507	0.20	79.82 (70.82–86.66)	39.74 (29.03–51.48)	1.32 (1.08–1.62)	0.51 (0.34–0.77)	64.93 (56.15–72.83)	58.49 (44.18–71.58)
4-lncRNA panel	0.89 (0.84–0.94)	>0.679	0.62	86.96 (77.94–92.79)	74.65 (62.69–83.90)	3.43 (2.28–5.15)	0.17 (0.10–0.30)	81.63 (72.26–88.47)	81.54 (69.59–89.70)

^#^The area under the curves of each lncRNA marker was compared with 4-lncRNA panel using the DeLong test with P<0.05. AUC, area under the curve; +LR, positive likelihood ratio; -LR, negative likelihood ratio; PPV, positive predictive value; NPV, negative predictive value; CI, confidence interval.

**Figure 4:**
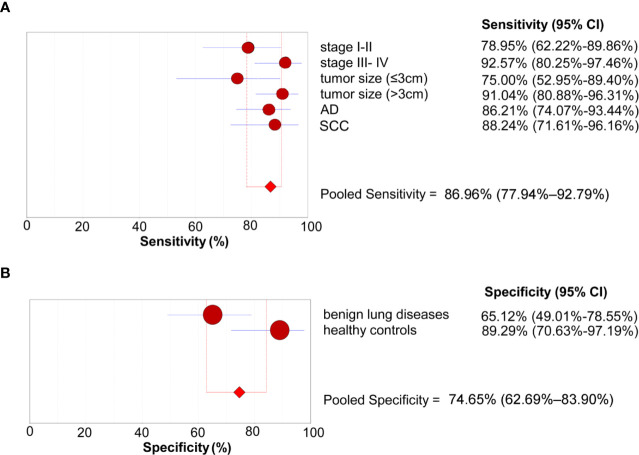
Subgroup analyses of the diagnostic performance of the 4-lncRNA panel in the verification phase. **(A)** Sensitivity (patients with lung cancer). **(B)** Specificity (control groups). AD, Adenocarcinoma; SCC, Squamous cell carcinoma.

We further conducted subgroup analyses to investigate the sensitivity of the 4-lncRNA panel in patients with different clinical stages, tumor size, or histological types. The sensitivities ranged from 75.00% to 92.57% in NSCLC patients, with a sensitivity of 78.95% (95% CI = 62.22%–89.86%) in stage I-II patients, 92.57% (95% CI = 80.25%–97.46%) in stage III- IV patients, 75.00% (95% CI = 52.95%–89.40%) in patients with small tumor size (≤3 cm), 91.04% (95% CI = 80.88%–96.31%) in patients with large tumor size (>3 cm), 86.21% (95% CI = 74.07%–93.44%) in lung adenocarcinoma patients, and 88.24% (95% CI = 71.61%–96.16%) in lung squamous cell carcinoma patients (**Figure 4A** **)**. We also conducted subgroup analyses to investigate the specificity of the 4-lncRNA panel in different control groups. The specificities ranged from 65.12% to 89.29% in control groups, with a specificity of 65.12% (95% CI = 49.01%–78.55%) in benign lung diseases, and 89.29% (95% CI = 70.63%–97.19%) in healthy controls (**Figure 4B** **)**. Furthermore, we evaluated the diagnostic value of 4-lncRNA panel in distinguishing lung cancer from specific benign diseases, such as COPD, pulmonary tuberculosis, and pulmonary inflammation. The 4-lncRNA panel also provided a good diagnostic capacity to distinguish NSCLC patients from COPD (AUC = 0.897, 95% CI = 0.825–0.947), pulmonary tuberculosis (AUC = 0.820, 95% CI = 0.733–0.888), or pulmonary inflammation (AUC = 0.897, 95% CI = 0.820–0.949) (**Supplementary Figure S2**).

### Comparison of the Diagnostic Performance of the 4-lncRNA Panel With Routine Protein Markers in the Expansion Phase

**Figure 5:**
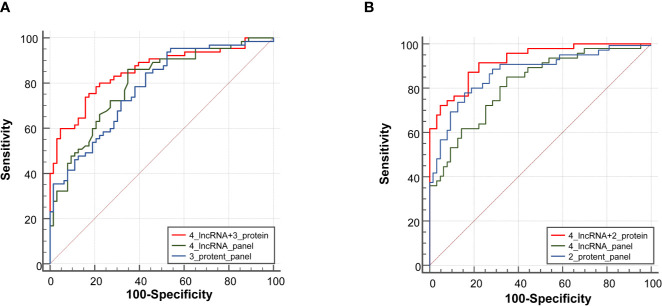
Receiver operating characteristic curve analysis for the diagnosis values of the 4-lncRNA panel and tumor protein panels. **(A)** Adenocarcinoma. **(B)** Squamous cell carcinoma.

In the expansion phase, a total of 240 participants who received the full panel of tumor marker test including CEA, CA125, CYFRA21-1, SCC, and NSE before therapy. Firstly, a predictive tumor marker panel was established by a stepwise logistic regression model using the expansion phase samples. CEA, CA125, and CYFRA21-1 were included in the regression model for adenocarcinoma, while SCC and CYFRA21-1 were included in the model of squamous cell carcinoma. The AUC value of the 3-protein panel (CEA+CA125+CYFRA21-1) in adenocarcinoma was 0.77 (95% CI = 0.70–0.83), which was lower than that of 4-lncRNA panel (**Figure 5A**, **Supplementary Table S5**). The sensitivity of 4-lncRNA panel was higher than that of 3-protein panel (86.30% vs. 73.96%), while the specificity of 4-lncRNA panel was lower than that of 3-protein panel (64.62% vs. 70.42%) (**Supplementary Table S5**). For squamous cell carcinoma, the AUC value of the 2-protein panel (SCC+CYFRA21-1) was 0.84 (95% CI = 0.76–0.89), which was similar to that of 4-lncRNA panel (**Figure 5B**, **Supplementary Table S6**). In addition, we found that combination of the 4-lncRNA panel with protein markers significantly improved the diagnostic performances in both adenocarcinoma (AUC = 0.85, 95% CI = 0.78–0.91) and squamous cell carcinoma (AUC = 0.93, 95% CI = 0.86–0.97) (**Figure 5**, **Supplementary Tables S5 and S6**).

## Discussion

In this study, we revealed that plasma RMRP, NEAT1, TUG1, and MALAT1 were potential circulating diagnostic biomarkers for diagnosing NSCLC. The 4-lncRNA panel established by the logistic regression model provided a high diagnostic value in NSCLC. We also compared its diagnostic value with tumor protein markers, and found that the 4-lncRNA panel had a markedly higher sensitivity in diagnosing NSCLC.

Current medical detection methods (including imaging and biomarker detection) for the diagnosis of cancer are emerging (19). However, the early diagnosis of lung cancer is still a great challenge (20). Circulating lncRNAs were stably expressed and were considered to be novel potential biomarkers to diagnose lung cancer (21, 22). In the training phase and verification phase, our study revealed that plasma RMRP, NEAT1, TUG1, and MALAT1 were potential circulating markers for diagnosing NSCLC. The 4-lncRNA panel showed a high accuracy in the diagnosis of NSCLC. Moreover, the 4-lncRNA panel had high specificity (76.05%–76.23%) while maintaining high sensitivity (85.26%–87.02%), indicating that the model had strong ability to detect NSCLC patients and could specifically exclude non-NSCLC patients. MALAT1 was firstly identified as a candidate circulating biomarker for the diagnosis of NSCLC (23). Subsequently, the diagnostic roles of circulating lncRNAs for NSCLC have been demonstrated in several studies. However, the diagnostic performances of circulating lncRNAs in different studies were inconsistent. For example, Guo et al. (24) reported that MALAT1 as a candidate blood-based biomarker to diagnosis lung cancer with an AUC value of 0.718. However, Liang et al. (13) found that plasma GAS5 expression level could be used to distinguish NSCLC patients from control patients with a relatively high AUC value of 0.832. Wang et al. (25) conducted a meta-analysis including 2121 NSCLC patients and 1,528 healthy controls and suggested miRNAs had a moderate diagnostic accuracy for lung cancer (sensitivity 75%, specificity 79%). Xie et al. (26) developed a diagnostic panel consisting of SOX2OT, ANRIL, CEA, CYFRA21-1, and SCCA, which could be valuable in NSCLC diagnosis (sensitivity = 77.1%, specificity = 79.2%). Tao et al. (27) detected the expression of exosomal lncRNAs in NSCLC and found that the combination of two exosomal lncRNAs had a similar diagnostic efficiency (sensitivity = 81.3%, specificity = 69.3%). In addition, Kamel et al. (28) demonstrated that the combination of GAS5 and SOX2OT showed a better diagnostic efficiency (sensitivity = 83.8%, specificity = 81.4%). The sensitivity of the 4-lncRNA panel model in our study is higher than the above studies, and the specificity is similar to the above studies. The reported inconsistent diagnostic lncRNA panels may be attributed to the differences in candidate lncRNAs profiles, specimen types (serum, plasma, and serum exosome), source of controls and patients. Although there are various methods of RNA isolation and qRT-PCR analysis used to detect the expression of circulating lncRNA, so far there is still no uniform standard. Thus, heterogeneity in RNA isolation and qRT-PCR methods could also be important reasons for the inconsistent findings.

Compared with those studies of circulating lncRNAs in diagnosing NSCLC (29–32), our study is unique for the following reasons: Firstly, we selected 31 lung cancer related lncRNAs based on the previous studies and screened the expression of these lncRNAs in the plasma, which make it easier to obtain effective diagnostic markers for lung cancer. Secondly, we included not only healthy controls but also benign lung diseases in the control group. Our subgroup analyses indicated that the 4-lncRNA panel also provided a good diagnostic capacity to distinguish NSCLC patients from COPD, pulmonary tuberculosis, or pulmonary inflammation.

We also confirmed the stability of 4 lncRNAs in plasma under harsh conditions. The reasons for the stability of lncRNAs in plasma could be that lncRNAs were encapsulated in some small vesicles (such as exosomes) (33, 34). In addition, lncRNAs could be folded into secondary and tertiary architectural domains and combined with proteins to form a complex which was protected from RNase degradation (35, 36). The stable expression of lncRNAs in plasma lay the foundation to act as diagnostic markers in NSCLC.

In clinical practice, tumor protein markers are widely used for screening and diagnosis of lung cancer (37–39). Thus, we further evaluated the diagnostic values of the 4-lncRNA panel for NSCLC by comparison with tumor protein markers. The 4-lncRNA panel showed a higher sensitivity than protein panel, in adenocarcinoma while the specificity of 4-lncRNA panel was lower than tumor protein markers. Therefore, we believe that the 4-lncRNA panel could supplement the lack of sensitivity of tumor protein markers. In fact, our additional analyses on the combinations of 4-lncRNA and protein markers showed a better diagnostic value in distinguishing lung adenocarcinoma or lung squamous cell carcinoma from controls.

In conclusion, we identified a plasma 4-lncRNA panel that distinguished NSCLC patients from healthy and benign lung diseases with a high degree of sensitivity and specificity. In addition, the 4-lncRNA panel could improve the diagnostic values of traditional tumor protein markers. Our plasma 4-lncRNA panel showed robust potential for the early diagnosis of NSCLC, suggesting circulating lncRNAs could be used as promising candidates for NSCLC diagnosis.

## Data Availability Statement

All the data generated for this study are included in the article/**Supplementary Material**.

## Ethics Statement

The studies involving human participants were reviewed and approved by the ethics committee of the Army Medical University (Chongqing, China). The patients/participants provided their written informed consent to participate in this study.

## Author Contributions

YL led the study by designing, conducting, interpreting results, writing the manuscript, and obtaining the funding. SY, YX, and XG performed the majority of the experiments and participated in the study design, result interpretation, and manuscript writing. YZ, CL, and LW collected human tissue samples and clinical data. WX and NW performed the statistical analysis. TC and XM contributed to the result interpretation and discussions. LB and ZY participated in the study design, participant recruitment, and result interpretation. All authors contributed to the article and approved the submitted version.

## Funding

This work was supported by the Chongqing Natural Science Foundation of China (No. cstc2019jcyj-msxmX0194 to Y. X.), the National Natural Science Foundation of China (No. 81871896, 81672316 and 81472190 to Y. L.; No. 81602933 to W. X.; No. 81370139 to L. B.).

## Conflict of Interest

The authors declare that the research was conducted in the absence of any commercial or financial relationships that could be construed as a potential conflict of interest.

## References

[1] Siegel RL Miller KD Jemal A Cancer statistics, 2018. CA Cancer J Clin (2018) 68(1):7–30. 10.3322/caac.21442 29313949

[2] Chen W Zheng R Baade PD Zhang S Zeng H Bray F Cancer statistics in China, 2015. CA Cancer J Clin (2016) 66(2):115–32. 10.3322/caac.21338 26808342

[3] Torre LA Siegel RL Jemal A Lung Cancer Statistics. Adv Exp Med Biol (2016) 893:1–19. 10.1007/978-3-319-24223-1_1 26667336

[4] Wood DE Kazerooni EA Baum SL Eapen GA Ettinger DS Hou L Lung Cancer Screening, Version 3.2018, NCCN Clinical Practice Guidelines in Oncology. J Natl Compr Canc Netw (2018) 16(4):412–41. 10.6004/jnccn.2018.0020 PMC6476336 29632061

[5] Veronesi G Novellis P Voulaz E Alloisio M Early detection and early treatment of lung cancer: risks and benefits. J Thorac Dis (2016) 8(9):E1060–2. 10.21037/jtd.2016.08.76 PMC5059339 27747063

[6] Snowsill T Yang H Griffin E Long L Varley-Campbell J Coelho H Low-dose computed tomography for lung cancer screening in high-risk populations: a systematic review and economic evaluation. Health Technol Assess (2018) 22(69):1–276. 10.3310/hta22690 PMC6304730 30518460

[7] Silva M Pastorino U Sverzellati N Lung cancer screening with low-dose CT in Europe: strength and weakness of diverse independent screening trials. Clin Radiol (2017) 72(5):389–400. 10.1016/j.crad.2016.12.021 28168954

[8] Villalobos P Wistuba II: Lung Cancer Biomarkers. Hematol Oncol Clin North Am (2017) 31(1):13–29. 10.1016/j.hoc.2016.08.006 27912828 PMC5137804

[9] Chatterjee M Sengupta S Emerging roles of long non-coding RNAs in cancer. J Biosci (2019) 44(1):22. 10.1007/s12038-018-9820-z 30837373

[10] Wei MM Zhou GB Long Non-coding RNAs and Their Roles in Non-small-cell Lung Cancer. Genomics Proteomics Bioinformatics (2016) 14(5):280–8. 10.1016/j.gpb.2016.03.007 PMC5093404 27397102

[11] Hu X Bao J Wang Z Zhang Z Gu P Tao F The plasma lncRNA acting as fingerprint in non-small-cell lung cancer. Tumour Biol (2016) 37(3):3497–504. 10.1007/s13277-015-4023-9 26453113

[12] Zhang R Xia Y Wang Z Zheng J Chen Y Li X Serum long non coding RNA MALAT-1 protected by exosomes is up-regulated and promotes cell proliferation and migration in non-small cell lung cancer. Biochem Biophys Res Commun (2017) 490(2):406–14. 10.1016/j.bbrc.2017.06.055 28623135

[13] Liang W Lv T Shi X Liu H Zhu Q Zeng J Circulating long noncoding RNA GAS5 is a novel biomarker for the diagnosis of nonsmall cell lung cancer. Med (Baltimore) (2016) 95(37):e4608. 10.1097/MD.0000000000004608 PMC5402552 27631209

[14] Lin Y Leng Q Zhan M Jiang F A Plasma Long Noncoding RNA Signature for Early Detection of Lung Cancer. Transl Oncol (2018) 11(5):1225–31. 10.1016/j.tranon.2018.07.016 PMC6089091 30098474

[15] Liu H Zhou G Fu X Cui H Pu G Xiao Y Long noncoding RNA TUG1 is a diagnostic factor in lung adenocarcinoma and suppresses apoptosis via epigenetic silencing of BAX. Oncotarget (2017) 8(60):101899–910. 10.18632/oncotarget.22058 PMC5731922 29254212

[16] Li N Wang Y Liu X Luo P Jing W Zhu M Identification of Circulating Long Noncoding RNA HOTAIR as a Novel Biomarker for Diagnosis and Monitoring of Non-Small Cell Lung Cancer. Technol Cancer Res Treat (2017) 16(6):1060–6. 10.1177/1533034617723754 PMC5762071 28784052

[17] Chen Q Zhu C Jin Y Si X Jiao W He W Plasma Long Non-Coding RNA RP11-438N5.3 as a Novel Biomarker for Non-Small Cell Lung Cancer. Cancer Manag Res (2020) 12:1513–21. 10.2147/CMAR.S237024 PMC7055527 32184656

[18] Li C Lv Y Shao C Chen C Zhang T Wei Y Tumor-derived exosomal lncRNA GAS5 as a biomarker for early-stage non-small-cell lung cancer diagnosis. J Cell Physiol (2019) 234(11):20721–7. 10.1002/jcp.28678 31032916

[19] Nimse SB Sonawane MD Song KS Kim T Biomarker detection technologies and future directions. Analyst (2016) 141(3):740–55. 10.1039/c5an01790d 26583164

[20] Tsiakkis D Graham Y Cox J Early diagnosis of lung cancer: is rapid access CT scanning the answer? Br J Gen Pract (2019) 69(679):90–1. 10.3399/bjgp19X701189 PMC6355293 30705018

[21] Chen X Dai M Zhu H Li J Huang Z Liu X Evaluation on the diagnostic and prognostic values of long non-coding RNA BLACAT1 in common types of human cancer. Mol Cancer (2017) 16(1):160. 10.1186/s12943-017-0728-2 29037201 PMC5644079

[22] Shi T Gao G Cao Y Long Noncoding RNAs as Novel Biomarkers Have a Promising Future in Cancer Diagnostics. Dis Markers (2016) 2016:9085195. 10.1155/2016/9085195 27143813 PMC4842029

[23] Weber DG Johnen G Casjens S Bryk O Pesch B Jöckel KH Evaluation of long noncoding RNA MALAT1 as a candidate blood-based biomarker for the diagnosis of non-small cell lung cancer. BMC Res Notes (2013) 6:518. 10.1186/1756-0500-6-518 24313945 PMC4029199

[24] Guo F Yu F Wang J Li Y Li Y Li Z Expression of MALAT1 in the peripheral whole blood of patients with lung cancer. Biomed Rep (2015) 3(3):309–12. 10.3892/br.2015.422 PMC4467267 26137228

[25] Wang H Wu S Zhao L Zhao J Liu J Wang Z Clinical use of microRNAs as potential non-invasive biomarkers for detecting non-small cell lung cancer: a meta-analysis. Respirology (2015) 20(1):56–65. 10.1111/resp.12444 25440223

[26] Xie Y Zhang Y Du L Jiang X Yan S Duan W Circulating long noncoding RNA act as potential novel biomarkers for diagnosis and prognosis of non-small cell lung cancer. Mol Oncol (2018) 12(5):648–58. 10.1002/1878-0261.12188 PMC5928376 29504701

[27] Tao Y Tang Y Yang Z Wu F Wang L Yang L Exploration of Serum Exosomal LncRNA TBILA and AGAP2-AS1 as Promising Biomarkers for Diagnosis of Non-Small Cell Lung Cancer. Int J Biol Sci (2020) 16(3):471–82. 10.7150/ijbs.39123 PMC6990900 32015683

[28] Kamel LM Atef DM Mackawy AMH Shalaby SM Abdelraheim N Circulating long non-coding RNA GAS5 and SOX2OT as potential biomarkers for diagnosis and prognosis of non-small cell lung cancer. Biotechnol Appl Biochem (2019) 66(4):634–42. 10.1002/bab.1764 31077615

[29] Huang Y Liu G Ma H Tian Y Huang C Liu F Plasma lncRNA FEZF1-AS1 as a potential biomarker for diagnosis of non-small-cell lung carcinoma. Med (Baltimore) (2020) 99(26):21019. 10.1097/MD.0000000000021019 PMC7329120 32590821

[30] Zhang X Guo H Bao Y Yu H Xie D Wang X Exosomal long non-coding RNA DLX6-AS1 as a potential diagnostic biomarker for non-small cell lung cancer. Oncol Lett (2019) 18(5):5197–204. 10.3892/ol.2019.10892 PMC6781719 31612030

[31] Li N Feng XB Tan Q Luo P Jing W Zhu M Identification of Circulating Long Noncoding RNA Linc00152 as a Novel Biomarker for Diagnosis and Monitoring of Non-Small-Cell Lung Cancer. Dis Markers (2017) 2017:7439698. 10.1155/2017/7439698 29375177 PMC5742528

[32] Jiang N Meng X Mi H Chi Y Li S Jin Z Circulating lncRNA XLOC_009167 serves as a diagnostic biomarker to predict lung cancer. Clin Chim Acta (2018) 486:26–33. 10.1016/j.cca.2018.07.026 30025752

[33] Hewson C Morris KV Form and Function of Exosome-Associated Long Non-coding RNAs in Cancer. Curr Top Microbiol Immunol (2016) 394:41–56. 10.1007/82_2015_486 26739961

[34] Akers JC Gonda D Kim R Carter BS Chen CC Biogenesis of extracellular vesicles (EV): exosomes, microvesicles, retrovirus-like vesicles, and apoptotic bodies. J Neurooncol (2013) 113(1):1–11. 10.1007/s11060-013-1084-8 23456661 PMC5533094

[35] Liu F Somarowthu S Pyle AM Visualizing the secondary and tertiary architectural domains of lncRNA RepA. Nat Chem Biol (2017) 13(3):282–9. 10.1038/nchembio.2272 PMC6432788 28068310

[36] Chen LL Linking Long Noncoding RNA Localization and Function. Trends Biochem Sci (2016) 41(9):761–72. 10.1016/j.tibs.2016.07.003 27499234

[37] Liu L Teng J Zhang L Cong P Yao Y Sun G The Combination of the Tumor Markers Suggests the Histological Diagnosis of Lung Cancer. BioMed Res Int (2017) 2017:2013989. 10.1155/2017/2013989 28607926 PMC5451759

[38] Chen ZQ Huang LS Zhu B Assessment of Seven Clinical Tumor Markers in Diagnosis of Non-Small-Cell Lung Cancer. Dis Markers (2018) 2018:9845123. 10.1155/2018/9845123 30647803 PMC6311751

[39] Jiang ZF Wang M Xu JL Thymidine kinase 1 combined with CEA, CYFRA21-1 and NSE improved its diagnostic value for lung cancer. Life Sci (2018) 194:1–6. 10.1016/j.lfs.2017.12.020 29247745

