# Supplementary material for: Circulating Long Noncoding RNAs Act as Diagnostic Biomarkers in Non-Small Cell Lung Cancer
Source: Front Oncol. 2020 Dec 7;10:537120. doi: 10.3389/fonc.2020.537120 (PMC7793881; doi:10.3389/fonc.2020.537120)
Supplement: Supplementary file 1 [file DataSheet_1.doc]

**Supplementary Table S1.** Primer sequences used in this study

| **LncRNA** | **Forward Primer sequence (5’-3’)** | **Reverse Primer sequence (5’-3’)** | **Length (bp)** |
| --- | --- | --- | --- |
| HOTAIR | GGAAAGATCCAAATGGGACCA | CTAGGAATCAGCACGAAGCAAA | 297 |
| MALAT1 | CTTCCCTAGGGGATTTCAGG | GCCCACAGGAACAAGTCCTA | 76 |
| CCAT2 | CCCTGGTCAAATTGCTTAACCT | TTATTCGTCCCTCTGTTTTATGGAT | 66 |
| H19 | GACTCAGGAATCGGCTCTGG | CTGCTGTTCCGATGGTGTCT | 164 |
| ANRIL | TTGTGAAGCCCAAGTACTGC | TTCACTGTGGAGACGTTGG | 146 |
| UCA1/CUDR | CTCTCCATTGGGTTCAC | GCGGCAGGTCTTAAGAGATGAG | 254 |
| SOX2OT | GCTCGTGGCTTAGGAGATTG | CTGGCAAAGCATGAGGAACT | 116 |
| MEG3 | TGTGGGATGTTGGTTTCGCC | CCACATTCGAGGTCCCTTCC | 204 |
| TUG1 | CTGAAGAAAGGCAACATC | GTAGGCTACTACAGGATTTG | 140 |
| SPRY4-IT1 | GCTGAGCTGGTGGTTGAAAGGAATC | GCTTGGCCCACGATGACTTGG | 130 |
| BANCR | CAAACCTGAATCTCACCTCTGC | TGCCAGGGATGACTTGCGTA | 76 |
| lncRNA-LET | AGGAGTCCTTGGACCTGAGC | AGTGGCTGGCATATAACCAACA | 116 |
| GAS5 | TCCCAGCCTCAGACTCAACA | GTTTCATAGGCCCCTGTGCT | 88 |
| LincRNA-p21 | CCCGGGCTTGTCTTTTGTT | GAGTGGGTGGCTCACTCTTCTG | 76 |
| lncRNA-MVIH | AATTTTGCACATCTGAACAGCC | TTCAAAATCCCACTACGCCCA | 79 |
| LINC01133 | GCTGTGGTGGAGAGAATGGA | CCCCAGCTTTCCAGATCCAAA | 93 |
| RMRP | ACTCCAAAGTCCGCCAAGA | TGCGTAACTAGAGGGAGCTGAC | 81 |
| PVT1 | TGAGAACTGTCCTTACGTGACC | AGAGCACCAAGACTGGCTCT | 74 |
| ATB | CTTCACCAGCACCCAGAGA | AAGACAGAAAAACAGTTCCGAGTC | 94 |
| lnc-TCF7 | AGGAGTCCTTGGACCTGAGC | AGTGGCTGGCATATAACCAACA | 116 |
| XIST | GCATAACTCGGCTTAGGGCT | TCCTCTGCCTGACCTGCTAT | 81 |
| NEAT1 | CTTCCTCCCTTTAACTTATCCATTCAC | CTCTTCCTCCACCATTACCAACAATAC | 116 |
| HOTTIP | CACACTCACATTCGCACACT | TCCAGAACTAAGCCAGCCATA | 156 |
| AGAP2-AS1 | TACCTTGACCTTGCTGCTCTC | TGTCCCTTAATGACCCCATCC | 187 |
| LINC00673 | TACCACACCCTTTCTTGCCC | ACACTGGCCTCTTTACACGG | 168 |
| CASC2 | GCACATTGGACGGTGTTTCC | CCCAGTCCTTCACAGGTCAC | 160 |
| PCAT-1 | TGAGAAGAGAAATCTATTGGAACC | GGTTTGTCTCCGCTGCTTTA | 156 |
| AFAP1-AS1 | TCGCTCAATGGAGTGACGGCA | CGGCTGAGACCGCTGAGAACTT | 96 |
| HNF1A-AS1 | TCAAGAAATGGTGGCTAT | GCTCTGAGACTGGCTGAA | 148 |
| PANDAR | TGCACACATTTAACCCGAAG | CCCCAAAGCTACATCTATGACA | 71 |
| HMlincRNA717 | TGGATGCTTACAAAGGACTGG | CTGCAATTACGGAAAGAGCTG | 173 |
| *β*-actin | CCTGGCACCCAGCACAAT | GGGCCGACTCGTCA TAC | 143 |

**Supplementary Table S2.** Expression levels of five lncRNAs in the discovery datasets

| **LncRNA** | **Expression level [M (P25, P75)]** | | **Z** | **P** |
| --- | --- | --- | --- | --- |
|  | **NSCLC（n=20）** | **Control (n=20)** |  |  |
| RMRP | 0.297 | 0.912 | -3.344 | **0.001** |
|  | (0.119-0.441) | (0.558-1.907) |  |  |
| NEAT1 | 1.753 | 1.085 | -2.276 | **0.022** |
|  | (1.240-3.249) | (0.689-1.883) |  |  |
| TUG1 | 0.516 | 1.292 | -3.196 | **0.001** |
|  | (0.297-0.815) | (0.664-2.158) |  |  |
| MALAT1 | 2.085 | 0.827 | -2.958 | **0.002** |
|  | (0.969-3.238) | (0.329-1.224) |  |  |
| H19 | 0.993 | 1.123 | -0.096 | 0.534 |
|  | (0.356-1.716) | (0.296-2.756) |  |  |

**Supplementary Table S3.** Expression levels of five lncRNAs in the training datasets

| **LncRNA** | **Expression level [M (P25, P75)]** | | **Z** | ***P*** |
| --- | --- | --- | --- | --- |
|  | **NSCLC（n=148）** | **Control (n=117)** |  |  |
| RMRP | 0.398 | 0.980 | -5.51 | **<0.001** |
|  | (0.186-0.859) | (0.472-1.963) |  |  |
| NEAT1 | 1.886 | 1.032 | -6.151 | **<0.001** |
|  | (1.139-3.037) | (0.678-1.607) |  |  |
| TUG1 | 0.518 | 0.807 | -3.183 | **0.001** |
|  | (0.344-0.892) | (0.415-1.659) |  |  |
| MALAT1 | 1.283 | 0.658 | -4.133 | **0.001** |
|  | (0.626-2.514) | (0.301-1.633) |  |  |
| H19 | 0.993 | 1.320 | -1.025 | 0.305 |
|  | (0.283-2.613) | (0.360-3.300) |  |  |

**Supplementary Table S4.** Expression levels of four lncRNAs in the validation datasets

| **LncRNA** | **Expression level [M (P25, P75)]** | | **Z** | ***P*** |
| --- | --- | --- | --- | --- |
|  | **NSCLC（n=148）** | **Control (n=117)** |  |  |
| RMRP | 0.356 | 1.121 | -6.671 | **<0.001** |
|  | (0.146-0.790) | (0.510-2.399) |  |  |
| NEAT1 | 1.796 | 0.956 | -7.019 | **<0.001** |
|  | (1.301-3.028) | (0.532-1.578) |  |  |
| TUG1 | 0.525 | 0.943 | -4.163 | **<0.001** |
|  | (0.319-0.796) | (0.515-2.200) |  |  |
| MALAT1 | 1.149 | 0.766 | -3.66 | **0.001** |
|  | (0.601-2.616) | (0.277-1.400) |  |  |

**Supplementary Table S5.** The diagnosis value of the 3-protein panel and 4-lncRNA panel in adenocarcinoma in the expansion dataset

| **Markers** | **AUC**  **(95% CI)** | **Youden index** | **Sensitivity (%) (95% CI)** | **Specificity (%) (95% CI)** | **+LR**  **(95% CI)** | **-LR**  **(95% CI)** |
| --- | --- | --- | --- | --- | --- | --- |
| CEA+CA125+  CYFRA21-1 | 0.77  (0.70-0.83) | 0.44 | 73.96  (63.83-82.14) | 70.42  (58.25-80.37) | 2.50  (1.71-3.65) | 0.37  (0.26-0.52) |
| 4-lncRNA panel | 0.80  (0.73-0.87) | 0.51 | 86.30  (75.79-95.88) | 64.62  (51.70-75.80) | 2.44  (1.73-3.43) | 0.21  (0.12-0.38) |
| 4-lncRNA+  3-protein | 0.85  (0.78-0.91) | 0.58 | 73.85  (61.23–83.61) | 84.13  (72.28–91.72) | 4.65  (2.59-8.36) | 0.31  (0.21-0.47) |

AUC, area under the curve; +LR, positive likelihood ratio; -LR, negative likelihood ratio; CI, confidence interval.

**Supplementary Table S6.** The diagnosis value of the 2-protein panel and 4-lncRNA panel in squamous cell carcinoma in the expansion dataset

| **Markers** | **AUC**  **(95% CI)** | **Youden index** | **Sensitivity (%) (95% CI)** | **Specificity (%) (95% CI)** | **+LR**  **(95% CI)** | **-LR**  **(95% CI)** |
| --- | --- | --- | --- | --- | --- | --- |
| SCC+CYFRA21-1 | 0.84  (0.76-0.89) | 0.52 | 81.67  (69.15-90.07) | 70.42  (58.25-80.37) | 2.76  (1.89-4.03) | 0.26  (0.15-0.45) |
| 4-lncRNA panel | 0.82  (0.73-0.88) | 0.48 | 79.16  (64.60-89.04) | 69.23  (56.41-79.77) | 2.57  (1.74-3.81) | 0.30  (0.17-0.53) |
| 4-lncRNA  +2-protein | 0.93  (0.86-0.97) | 0.70 | 87.23  (73.56-94.70) | 82.53  (70.48-90.56) | 5.00  (2.89-8.64) | 0.15  (0.07-0.33) |

AUC, area under the curve; +LR, positive likelihood ratio; -LR, negative likelihood ratio; CI, confidence interval.

**
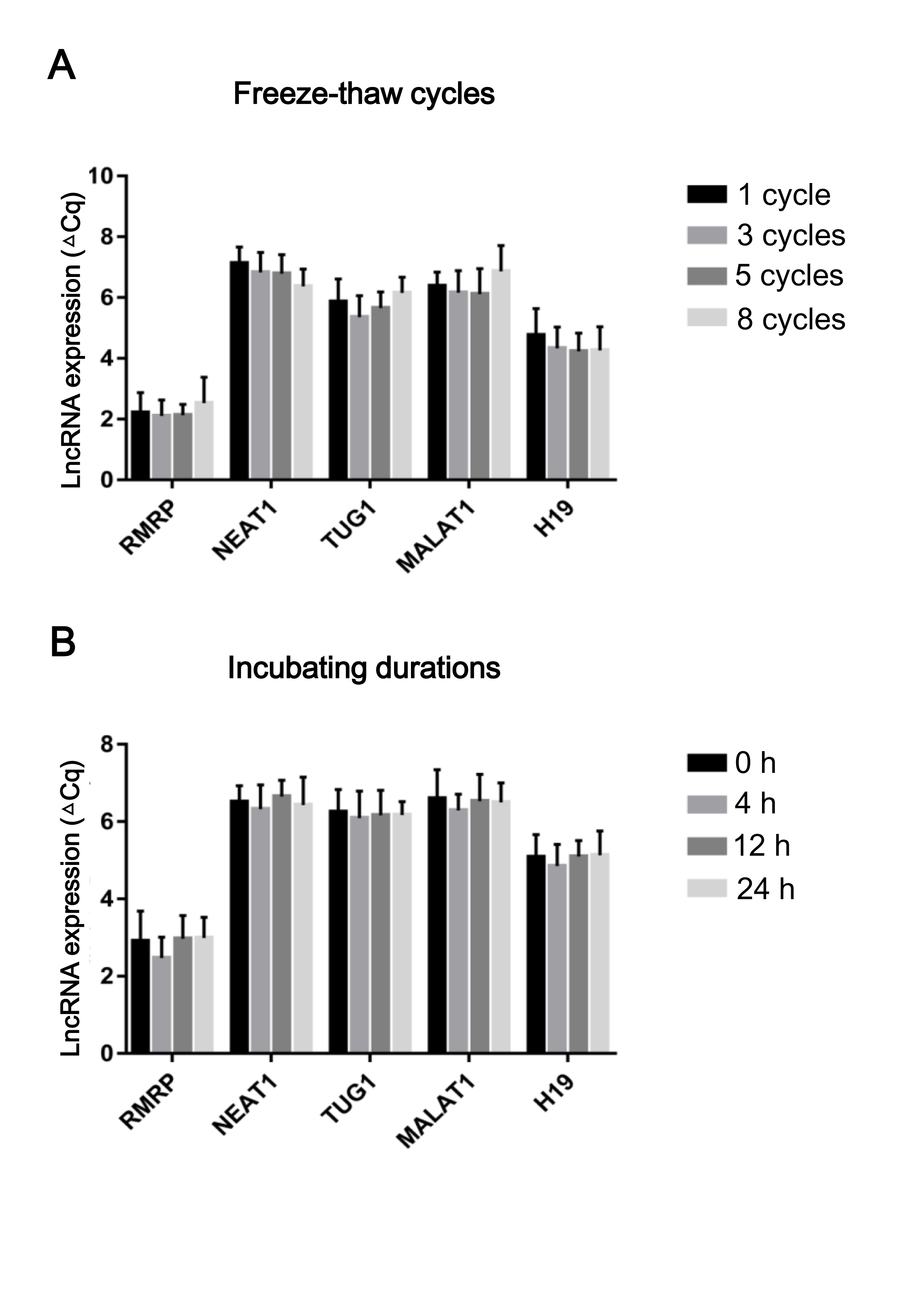
**

**Figure S1.** **The stability of RMRP, NEAT1, TUG1, MALAT1 and H19 in plasma under harsh conditions.** (A) The fresh plasma samples from 5 patients with lung cancer were treating with 1, 3, 5, 8 repetitive freeze-thaw cycles. (B) The fresh plasma samples from 5 patients were incubated for various durations (0, 4, 12, and 24 h) at room temperature. No significant difference was observed in each group (P > 0.05).


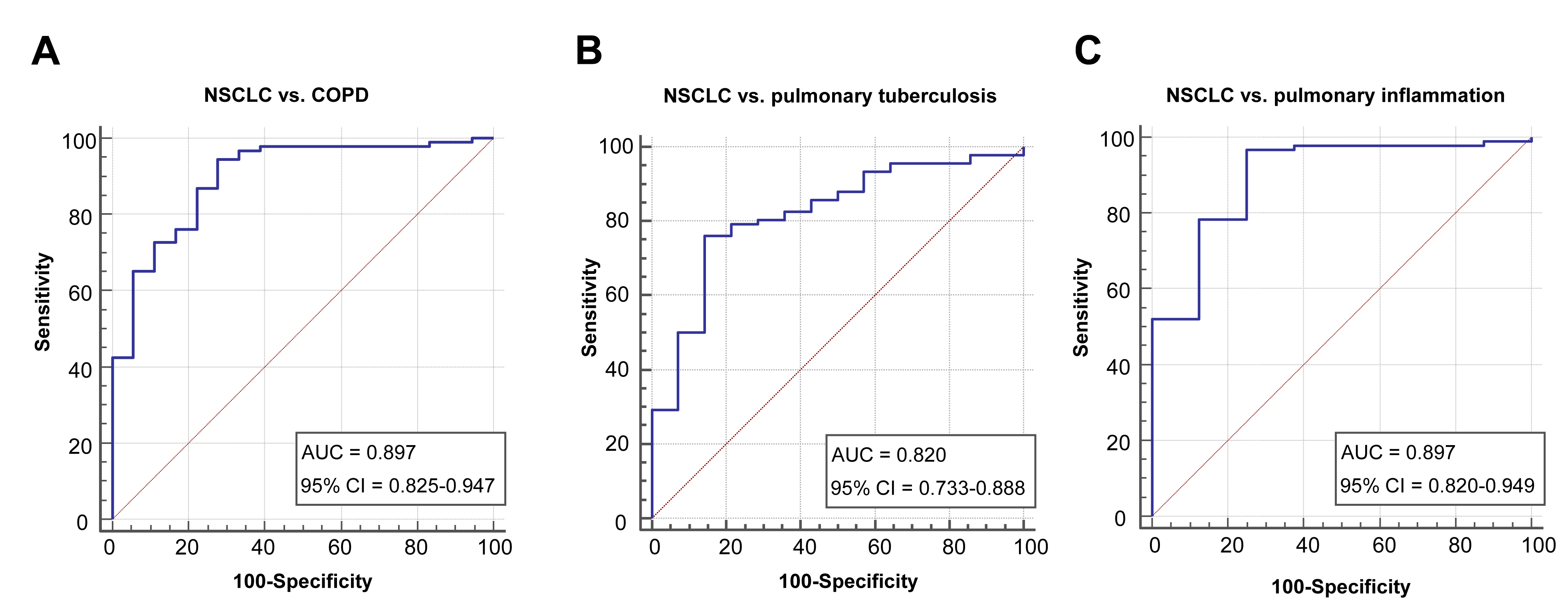


**Figure S2.** **Receiver operating characteristic curve analysis for the diagnosis values of the 4-lncRNA panel in distinguishing lung cancer from specific benign diseases.** (A) NSCLC vs. COPD. (B) NSCLC vs. pulmonary tuberculosis. (C) NSCLC vs. pulmonary inflammation.
